# TLR7/TLR8 Activation Restores Defective Cytokine Secretion by Myeloid Dendritic Cells but Not by Plasmacytoid Dendritic Cells in HIV-Infected Pregnant Women and Newborns

**DOI:** 10.1371/journal.pone.0067036

**Published:** 2013-06-27

**Authors:** Elaine Cristina Cardoso, Nátalli Zanete Pereira, Gabrielle Eimi Mitsunari, Luanda Mara da Silva Oliveira, Rosa Maria S. A. Ruocco, Rossana Pulcineli Vieira Francisco, Marcelo Zugaib, Alberto José da Silva Duarte, Maria Notomi Sato

**Affiliations:** 1 Laboratory of Dermatology and Immunodeficiencies, Department of Dermatology, Medical School, University of São Paulo, São Paulo, São Paulo, Brazil; 2 Hospital das Clínicas, Department of Obstetrics and Gynecology, Medical School, University of São Paulo, São Paulo, São Paulo, Brazil; University of California San Francisco, United States of America

## Abstract

Mother-to-child transmission (MTCT) of HIV-1 has been significantly reduced with the use of antiretroviral therapies, resulting in an increased number of HIV-exposed uninfected infants. The consequences of HIV infection on the innate immune system of both mother-newborn are not well understood. In this study, we analyzed peripheral blood and umbilical cord blood (CB) collected from HIV-1-infected and uninfected pregnant women. We measured TNF-α, IL-10 and IFN-α secretion after the stimulation of the cells with agonists of both extracellular Toll-like receptors (TLRs) (TLR2, TLR4 and TLR5) and intracellular TLRs (TLR7, TLR7/8 and TLR9). Moreover, as an indicator of the innate immune response, we evaluated the responsiveness of myeloid dendritic cells (mDCs) and plasmacytoid DCs (pDCs) to TLRs that are associated with the antiviral response. Our results showed that peripheral blood mononuclear cells (PBMCs) from HIV-1-infected mothers and CB were defective in TNF-α production after activation by TLR2, TLR5, TLR3 and TLR7. However, the TNF-α response was preserved after TLR7/8 (CL097) stimulation, mainly in the neonatal cells. Furthermore, only CL097 activation was able to induce IL-10 and IFN-α secretion in both maternal and CB cells in the infected group. An increase in IFN-α secretion was observed in CL097-treated CB from HIV-infected mothers compared with control mothers. The effectiveness of CL097 stimulation was confirmed by observation of similar mRNA levels of interferon regulatory factor-7 (IRF-7), IFN-α and TNF-α in PBMCs of both groups. The function of both mDCs and pDCs was markedly compromised in the HIV-infected group, and although TLR7/TLR8 activation overcame the impairment in TNF-α secretion by mDCs, such stimulation was unable to reverse the dysfunctional type I IFN response by pDCs in the HIV-infected samples. Our findings highlight the dysfunction of innate immunity in HIV-infected mother-newborn pairs. The activation of the TLR7/8 pathway could function as an adjuvant to improve maternal-neonatal innate immunity.

## Introduction

The main source of pediatric HIV-1 infection is mother-to-child transmission MTCT. In 2011, approximately 330,000 children were infected with HIV, representing a 43% decline since 2003 [1]. Highly effective strategies have been introduced to prevent the spread of HIV from mothers to their infants, resulting in an increased number of HIV-exposed uninfected infants [2]. These babies are at a greatly increased risk of death during the first year of life and appear to suffer from a weakness in their immune defenses [3], [4], [5], [6].

In the absence of treatment, 55 to 80% of infants exposed to HIV-1 remain uninfected [7]. There is a possibility that children escape infection because they are not exposed to enough virus, as cell-to-cell HIV-1 dissemination at the maternal-fetal interface appears to be tightly controlled [8]. Alternatively, children might escape infection due to the *in utero* development of HIV-1-specific T cell responses, a scenario that suggests that there was exposure to enough replicating virus to prime such responses [9], [10], or due to the generation of *in utero* CD4^+^CD25^+^ T regulatory cells that contribute to a reduction in T cell activation [11]. Furthermore, multiple mechanisms during pregnancy that regulate the development of an allogeneic fetus [12], [13] could be an appropriate microenvironment to prevent T cell activation in HIV-1 infection.

The proportion of uninfected children exposed to infected mothers is increasing and has compelled the need to understand the mechanisms of natural immunity and cross talk between the innate and adaptive immune responses to control HIV-1 infection. Moreover, considering that these infants are susceptible to infections [4] and that their immune systems are relatively immature [14], strategies to enhance the newborn immune response should be considered.

The innate immune system is the first line of defense and consists of cells that are able to recognize and respond to infections quickly through pattern recognition receptors (PRRs). The PRRs include Toll-like receptors (TLRs 2–9) that recognize conserved motifs unique to microorganisms and viruses, as well as dsRNA and ssRNA [15]. It has been shown that HIV-1 ssRNA encodes for multiple TLR7/TLR8 ligands that can mediate direct activation of the immune system *in vitro* [16], [17]. TLRs are expressed by various cell types, and stimulation of these receptors induces a complex network of signals important for dendritic cells (DCs) and for developing T and B cell adaptive responses [18], [19].

The neonatal immune system is relatively immature and highly plastic, but a mature response can be achieved upon innate stimulation [14], [20], [21]. Using cord blood (CB) as a source of immune cells, reports have indicated that the response of neonatal monocytes and DCs to TLR agonists shows a marked polarization towards the impaired production of Th1 cytokines and increased production of Th2/Th17 cytokines [22], [23], [24]. Moreover, TLR8 agonists are uniquely efficacious in activating costimulatory responses in neonatal DCs, suggesting that these agents are promising candidate adjuvants for enhancing immune responses in human newborns [25].

Human myeloid DCs (mDCs) express all TLRs except TLR7 and TLR9; the latter are selectively expressed by plasmacytoid DCs (pDCs) and recognize single-stranded RNA from HIV-1. TLR7 activation leads to the pDC production of type I IFN and other inflammatory cytokines [26], [27]. Exposure to HIV-1 *in utero* induces changes in neonatal DCs, particularly mDCs, which might be associated with the alterations observed in T cells of HIV-exposed uninfected children [28].

In the current study, we evaluated innate immune profiles following TLR stimulation in HIV-1-infected mothers and newborns. We found significantly compromised cytokine responses upon extracellular and intracellular TLR activation. Furthermore, mDC responsiveness to TLR activation appeared to be less impaired than the type I IFN response by pDCs in the peripheral blood and CB of HIV-infected mothers. Our findings highlight the complexities underlying adjuvant activation of the innate immune system and suggest that mDC function can be enhanced through TLR7/TLR8 activation.

## Results

### Cytokine Secretion in Response to TLR Stimulation in Peripheral Blood and CB Cells

Given the importance of innate immunity in the early phase of life, we assessed TLR activation by measuring cytokine secretion in blood taken from HIV-1-infected mothers and uninfected controls (Table 1) at the time of delivery and in umbilical CB. We assessed TNF-α, IL-10 and IFN-α secretion by PBMCs stimulated with agonists for extracellular TLRs (TLR2, TLR4 and TLR5) and intracellular TLRs (TLR3, TLR7, TLR7/8 and TLR9).

**Table 1:** Demographic characteristics of mothers.

	Mothers infectedwith HIV	Uninfected mothers
	N = 15	N = 15
**Age**	29.5 (17–35)	28 (18–33)
**CD4 mm^3^**	376 (96–376)	
**CD4 mm^3^ nadir**	406.5 (155–856)	
**CD8 mm^3^**	633 (250–1055)	
**Viral load copies/mL**	<50 (<50–111)	

Median (range).

**Figure 1:**
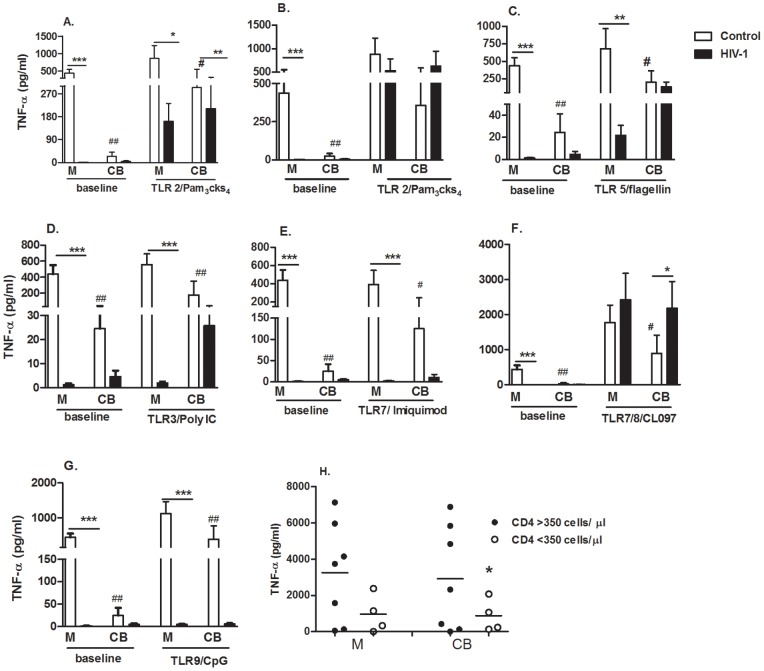
Profile of the TNF-α response induced by extracellular and intracellular TLR activation in peripheral blood and cord blood of HIV-1-infected mothers. (A–G) PBMCs obtained from mothers (M) and cord blood (CB) of HIV-1-infected mothers (n = 15) and uninfected controls (n = 15) were stimulated or not (baseline) for 48 h with agonists of extracellular TLRs (TLR2/Pam3Cks4, TLR4/LPS, TLR5/flagellin) or intracellular TLRs (TLR3/poly I:C, TLR7/Imiquimode, TLR7/TLR8/CL097, TLR9/CpG). Cell-free supernatants were assessed for TNF-α secretion by cytometric bead array. Bars indicate the mean ± SEM. *p≤0.05, **p≤0.01, ***p≤0.001 when compared with the control group; #p≤0.05, ##p≤0.01 when compared with the respective mother. (H) TNF-α secretion –induced by CL097 from HIV-infected mothers was assessed according to the mother CD4+ T cells number, <350 cells/µL (open circles) or >350 cells/µL (closed circles). Figure represents the median. *p≤0.05 when compared with the CD4+ T cells >350 cells/µL group.

Figure 1 shows that baseline TNF-α secretion by PBMCs was barely detectable in HIV-1-infected mothers compared with healthy controls. The activation of TLR2 using the Pam3Cks4 ligand induced TNF-α secretion by CB cells from HIV-1-infected mothers, but these levels were decreased compared with the healthy group. A similar ability to secrete TNF-α following LPS/TLR4 activation was detected in peripheral blood and CB from both HIV-infected and uninfected groups. Upon TLR5 activation using flagellin, HIV-infected mothers also showed impaired TNF-α secretion compared with control mothers. Moreover, CB cells secreted similar amounts of TNF-α as their adult counterparts.

TLRs associated with the antiviral response were assessed using the agonists poly I:C/TLR3, Imiquimod/TLR7, CL097/TLR7/8 and CpG/TLR9. For all ligands except CL097, we observed diminished TNF-α secretion in HIV-1-infected mothers compared with control mothers (Figure 1). Of note, CL097 induced enhanced TNF-α production in newborns from infected mothers (Figure 1F). Curiously, the ligand that activates both TLR7 and TLR8 enhanced TNF-α secretion in HIV-1 infected mothers a thousand times more compared with the ligand that only activates TLR7. CL097 compound highly induced TNF-α secretion in CB from HIV-1-infected mothers in comparison with CB from control mothers.

Because of the ability of CL097 to enhance TNF-α secretion, we next wanted to correlate TNF-α secretion with maternal CD4+ T cell counts. In naïve HIV-infected individuals, a CD4+ T cell count of <350 cells cells/µL is used as the criteria for beginning HAART therapy. Additionally, a CD4+ T cell count of >350 cells/µL in HAART-treated individuals can indicate CD4+ T cell reconstitution. We analyzed CL097-induced TNF-α in HIV-infected mothers with >350 CD4+ T cells/µL compared to infected mothers with <350 CD4+ T cells/µL (Figure 1H). TNF-α secretion was observed in infected mothers with >350 CD4+ T cells/µL, but this increase in secretion was not statistically significant compared to that observed in infected mothers with <350 CD4+ T cells/µL. However, by analyzing the secretion of TNF-α induced by CL097 in CB cells, we verified that infected mothers with a lower CD4+ T cell count have reduced TNF-α secretion. A relationship between TNF-α secretion and CD4+ T cell number was not found with any other TLR agonists analyzed.

**Figure 2:**
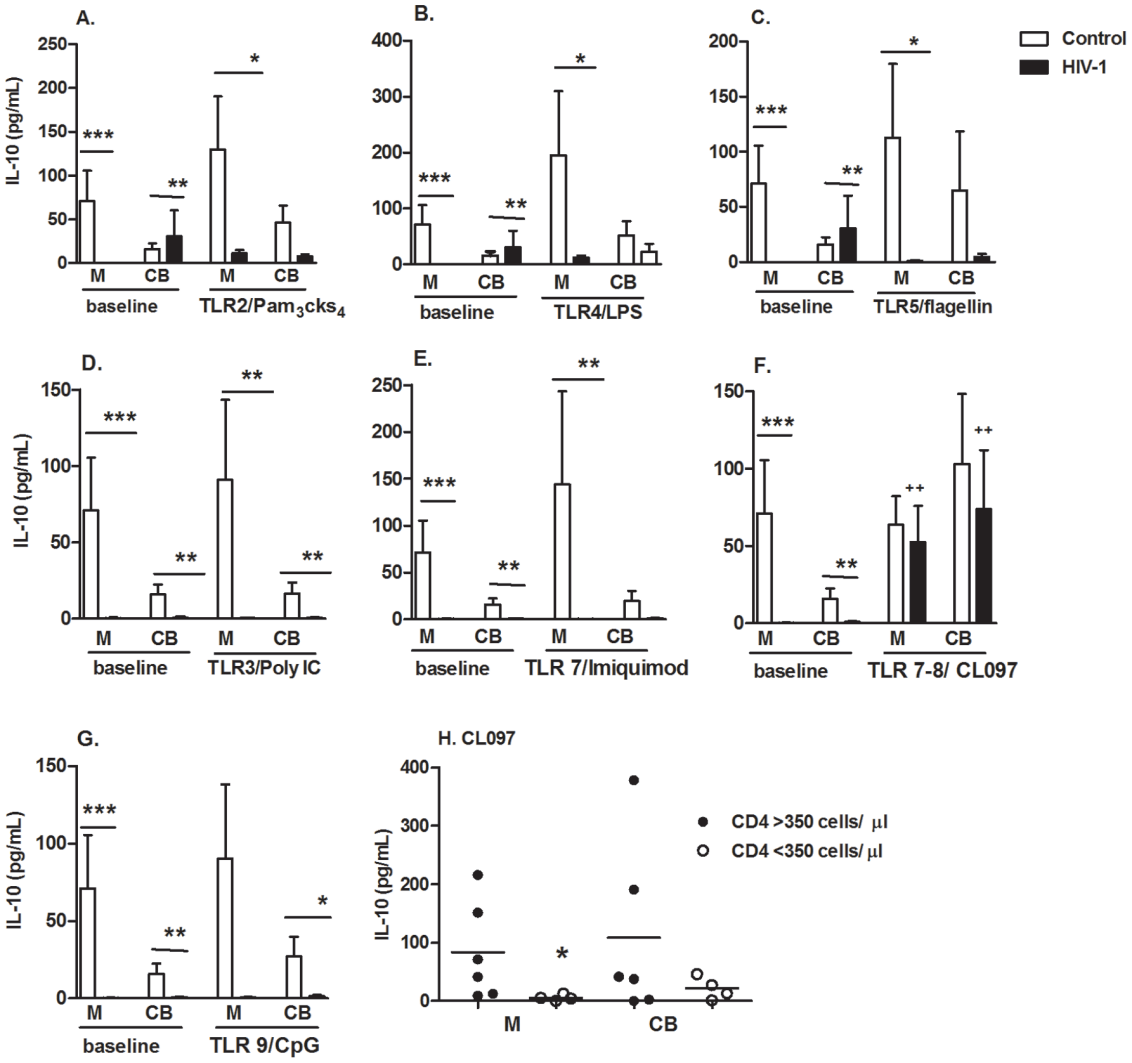
Enhanced IL-10 secretion by TLR7/8 ligand stimulation in HIV-1-infected mothers and newborns. (A–G) PBMCs from mothers (M) and cord blood (CB) of HIV-1-infected (n = 15) and uninfected controls (n = 15) were stimulated or not (baseline) for 48 h with agonists for TLR2/Pam3Cks4, TLR4/LPS, TLR5/flagellin, TLR3/poly I:C, TLR7/Imiquimode, TLR7/TLR8/CL097 and TLR9/CpG. IL-10 secretion was determined by cytometric bead array. Bars indicate the mean ± SEM. *p≤0.05, **p≤0.01, *** p≤0.001 when compared with the control group; ++p≤0.01 when compared with baseline levels. (H) IL-10 secretion –induced by CL097 from HIV-infected mothers was assessed according to the mother CD4+ T cells number, <350 cells/µL (open circles) or >350 cells/µL (closed circles). Figure represents the median. *p≤0.05 when compared with the CD4+ T cells >350 cells/µL group.

IL-10 secretion induced by extracellular and intracellular TLR agonist stimulation was significantly impaired in the infected mothers and CB compared with the uninfected controls (Figure 2). However, CL097 activation induced IL-10 secretion in the infected group, both in mothers and CB cells. We then evaluated CL097-induced IL-10 secretion in HIV-infected mothers, according CD4+ T cell counts dividing in >350 cells/µL and those with CD4+ T cell counts <350 cells/µL (Figure 2H). We found that only mothers with CD4+ T cell counts >350 cells/µL responded with IL-10 production.

**Figure 3:**
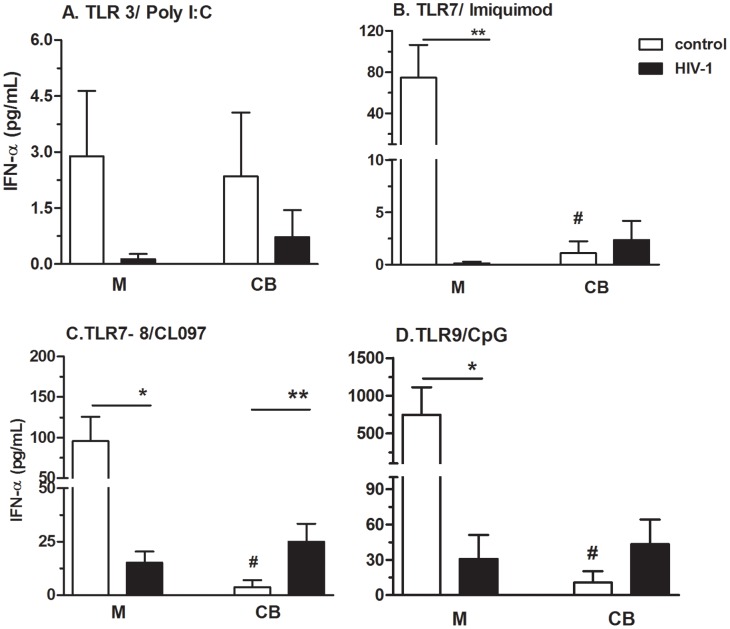
Newborns from HIV-infected mothers have enhanced IFN-α responses following TLR7/8 activation. PBMCs from mothers (M) and cord blood (CB) of HIV-1-infected (n = 15) and uninfected controls (n = 15) were stimulated or not (baseline) for 48 h with agonists of TLR3/poly I:C, TLR7/Imiquimode, TLR7/TLR8/CL097 and TLR9/CpG. IFN-α secretion was determined by cytometric bead array. Bars indicate the mean ± SEM. *p≤0.05, **p≤0.01 when compared with the control group; #p≤0.05 when compared with the mother.

We assessed IFN-α secretion as an indicator of the antiviral response upon TLR activation. As expected, extracellular TLR agonists did not trigger an IFN-α response, whereas intracellular TLR activation did (Figure 3). HIV-1-infected mothers showed a significant impairment in IFN-α secretion induced by TLR7/8/9 activation compared with control mothers. However, IFN-α levels in CB from HIV-infected mothers treated with CL097 were increased relative to CB from control mothers (Figure 3C).

We found that CL097-induced IL-10 secretion was positively correlated with CL097-induced TNF-α secretion in HIV-infected mothers and no correlation was observed in the control mothers (Figure S1). When we analyzed whether other cytokines could be correlated with a different agonist in HIV-infected mothers, we observed a significant positive correlation between TNF-α induced by flagellin/TLR5 and Pam3Cks/TLR2 (Figure S1).

The results show that peripheral blood and CB from HIV-1-infected mothers have an impaired cytokine response following intracellular TLR agonist activation but a preserved response upon TLR7/8 activation, and this rescued response occurred mainly in the CB cells.

### Expression of IRF-7, IFN-α and TNF-α in Response to TLR Stimulation in Peripheral Blood and CB Cells

**Figure 4:**
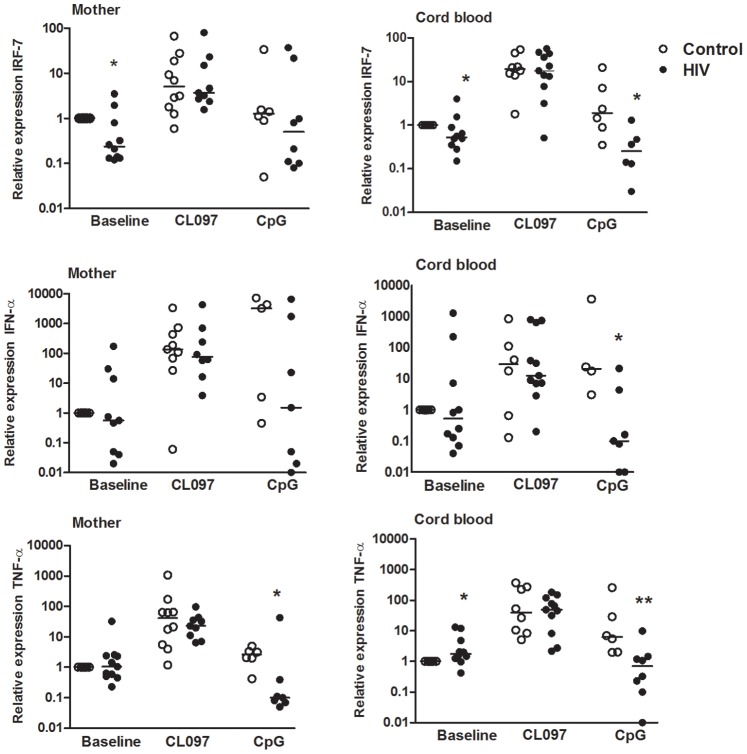
Profile of IRF-7, IFN-α and TNF-α gene expression induced by TLR7/8 and TLR9 activation. PBMCs from mothers (M, left side column) and cord blood (CB, right side column) of HIV-1-infected (n = 6–10, closed circles) and uninfected controls (n = 4–11, open circles) were stimulated or not (baseline) for 4 h with agonists of TLR7/TLR8 (CL097) and TLR9 (CpG). The expression of IRF-7, IFN-α and TNF-α were assessed by real–time PCR, and relative expression was normalized to GAPDH mRNA levels. *p≤0.05, **p≤0.01 when compared with the control group.

We next evaluated the expression of the transcription factor, IRF-7, as the production of IFN-α depends on this factor. Peripheral blood and CB cells were stimulated with CL097/TLR7/8 or CpG/TLR9 for 4 hours. Unstimulated PBMCs showed low IRF-7 expression in the infected group compared with the control group, but upon CL097 activation, the expression of IRF-7 and those of IFN-α and TNF-α were similar between the groups (Figure 4). Thus, the CL097 agonist restores the expression of IRF-7, IFN-α and TNF-α in both infected mothers and CB and consequently overcomes the defective TNF-α secretion, but CL097 rescues IFN-α only in CB cells (Figure 3C).

After stimulation with CpG, IRF-7, IFN-α and TNF-α mRNA expression was impaired in CB cells. In infected mothers, this impairment was not statistically significant for IRF-7 and IFN-α but was significant for TNF-α (Figure 4).

### Impaired IFN**-α** Secretion by DCs upon TLR Agonist Stimulation in HIV-1-Infected Mothers-CB

Subtypes of DCs, namely mDCs and pDCs, have been shown to have functional differences between neonates and adults that account for the characteristics of CB cells [29], [30], [31]. The relative immaturity of DCs could be an essential factor in the susceptibility to viral infection in neonates.

Therefore, we determined the key cytokines secreted by mDCs and pDCs upon TLR7, TLR7/8 and TLR9 activation in CB and peripheral blood from HIV-1 infected mothers. Strategies to identify DC subsets are shown in Figure S2. Under baseline conditions, we analyzed the mDC:pDC ratio in the mother’s blood and in the CB (Figure S3). There was a decreased mDC:pDC ratio in infected mothers compared to control mothers, which was caused by an increase in the percentage of pDCs. Moreover, a decreased mDC:pDC ratio was detected in the cord blood from infected mothers.

**Figure 5:**
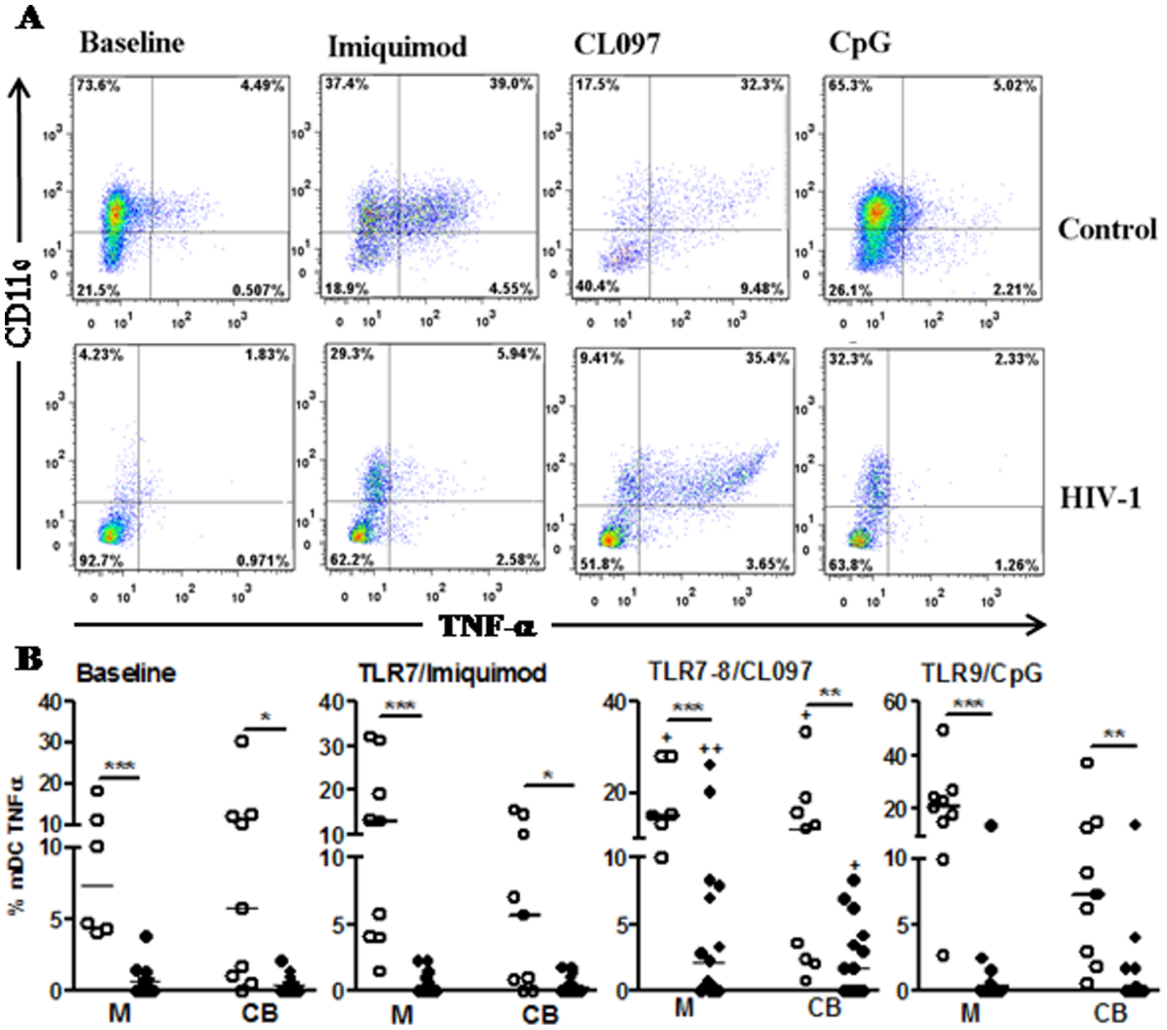
Improved TNF-α secretion by mDCs in peripheral blood and CB of HIV-1-infected mothers after stimulation with TLR7/8 agonists. PBMCs from mothers (M) and cord blood (CB) of HIV-1-infected (n = 15) and uninfected controls (n = 15) were stimulated or not (baseline) for 20 h with agonists of TLR7 (Imiquimode), TLR7/TLR8 (CL097) and TLR9 (CpG). Brefeldin was added after 5 h of stimulation. Intracellular TNF-α secretion by mDCs (CD11c+lin-HLA-DR+) was assessed by flow cytometry. a) representative histograms of mDCs (CD11c+TNF-α+) upon TLR stimulation. The number in the corner represents the cell percentage. b) Data represent the mean percentage ± SEM. *p≤0.05, **p≤0.01, ***p≤0.001 when compared with the control group; +p≤0.05, ++p≤0.01 when compared with baseline levels.

Figure 5 shows that the baseline percentage of mDCs secreting TNF-α was decreased in HIV-1-infected mothers and their neonatal counterparts compared with controls. Similar percentages of mDCs secreting TNF-α were detected between mothers and CB in both the infected and uninfected groups. No response to TLR7 or TLR9 ligands was observed in mDCs from peripheral blood and CB of HIV-1-infected mothers. In contrast, activation with the TLR7/8 agonist significantly increased TNF-α production by mDCs in both mothers’ blood and CB, although the percentage of mDCs secreting TNF-α remained lower in the HIV-1-infected mothers and CB than in the uninfected group.

**Figure 6:**
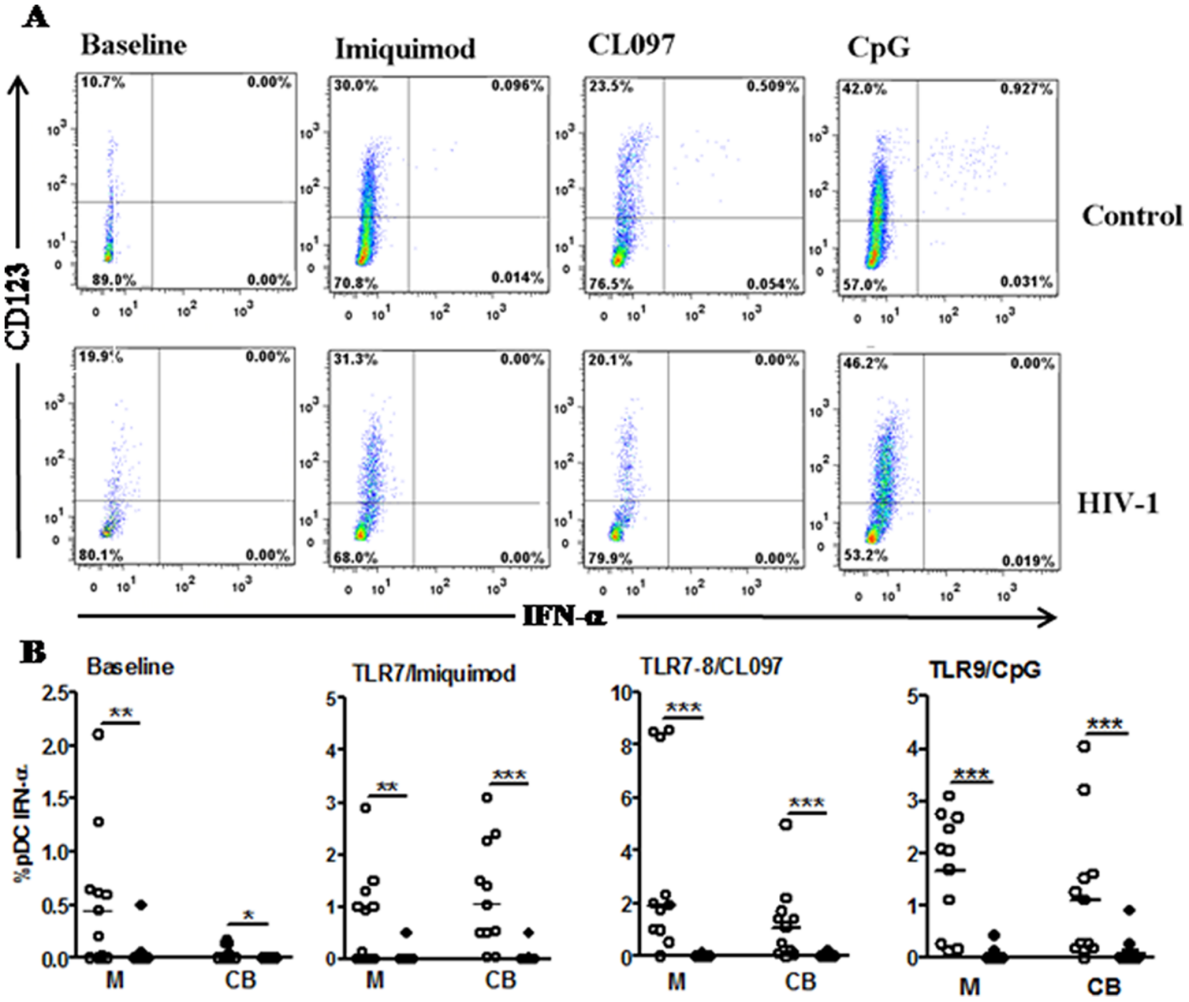
Marked decrease in pDC responsiveness to TLR activation in peripheral blood and CB from HIV-1-infected mothers. PBMCs from mothers (M) and cord blood (CB) of HIV-1-infected (n = 15) and uninfected controls (n = 15) were stimulated or not (baseline) for 20 h with agonists of TLR7 (Imiquimode), TLR7/TLR8 (CL097) and TLR9 (CpG). Brefeldin was added after 5 h of stimulation. Intracellular IFN-α secretion by pDCs (CD123+lin-HLA-DR+) was assessed by flow cytometry. a) Representative histograms of CD123+IFN-α+ cells upon TLR stimulation from one HIV-infected and one uninfected mother. The number in the corner represents the cell percentage. b) Data represent the mean percentage ± SEM. *p≤0.05, **p≤0.01, ***p≤0.001 when compared with the control group; +p≤0.05, ++p≤0.01, +++p≤0.001 when compared with baseline levels.

The baseline percentage of pDCs secreting IFN-α was also significantly decreased in infected mothers and CB compared with the control mothers (Figure 6). After TLR ligand stimulation, a barely detectable IFN-α response was observed in pDCs from HIV-infected mothers and CB compared with control mothers. In contrast, pDCs in the CB of control mothers showed a significantly higher IFN-α response upon activation with Imiquimode, CpG and CL097, thereby emphasizing the impaired response to TLR activation by mothers infected with HIV.

Our results show an important impairment of mDCs and pDCs and demonstrated that CL097 could overcome the impairment of mDC function in HIV-1-infected mothers and newborns.

## Discussion

The transplacental transmission of HIV is the result of a complex interaction between viral characteristics, and genetic, immunologic factors from mother and infant. Although profound damage to the adaptive immune system occurs during chronic HIV infection, other components of the immune system, including the innate immune response, can be affected [32]. The degree of impairment of the innate immune response in HIV-1-infected mothers and newborns following suppression of HIV-1 replication by antiretroviral therapy remains unclear. A better understanding of the immune development of these infants is crucial.

Our findings showed that HIV-infected mothers and newborns had a decreased TNF-α response upon extracellular TLR stimulation, including agonists for TLR2 (Pam3Cks4) and TLR5 (flagellin), and for intracellular TLR activation associated with the antiviral response, including TLR3 (poly I:C), TLR7 (imiquimod) and TLR9 (CpG). A possible explanation for the pronounced decrease in TLR responsiveness could be because chronic HIV-1 infection disturbs the active immunoregulatory mechanisms operating in pregnancy that maintain the semi-allogeneic fetus [12], [33]. Normally, increased TLR responsiveness occurs in viremic HIV infection, thereby augmenting the proinflammatory response to TLR ligands [34]. HIV-1-specific TLR ligands may directly contribute to the immune activation observed during viremic HIV-1 infection. In our study, all HIV positive mothers underwent HAART treatment and had undetectable viral loads and nadir CD4 T counts of 406.5 cells/µL during gestation. The low responsiveness of infected mothers to TLR ligands may suggest a dominance of pregnancy-associated tolerance mechanisms to avoid inflammatory exacerbation.

However, we found a preserved response upon stimulation with TLR4 (LPS) and TLR7/8 (CL097) agonists in PBMCs from HIV-infected mother-newborn pairs. TLR4 sensitivity could be partially due to immune activation of mother-newborn pairs, considering that HAART is known to reduce immune activation [35], [36], which has been ascribed to intestinal microbial translocation during HIV-1 infection [37], [38].

Interestingly, newborns from HIV-infected mothers had TNF-α responsiveness equal to their adult counterparts and enhanced responses to the TLR7/8 agonist compared with newborns from control mothers. In fact, human newborns have been shown to have a selective impairment in TLR activation and TNF-α release in response to bacterial lipopeptides, lipopolysaccharide and the imidazoquinoline compound, imiquimod [25], [39]. However, a response was generated for R-848, a TLR7/8 ligand, that was able to induce an equivalent response between CB and adult blood; this effect was specifically due to TLR8 activation [25], [39]. TLR8 agonists activate costimulatory responses in neonatal APCs [25], and this activity may represent an opportunity to augment innate and acquired immunity in the human newborn.

A response to TLR7/8 agonists in newborns from HIV-infected mothers was also observed in the form of IL-10 secretion. In the peripheral blood and CB of mothers infected with HIV, only low levels of IL-10 were released in response to extracellular or intracellular TLR agonists, but TLR7/8 ligands induced IL-10 production. This IL-10 response to TLR7/8 ligands demonstrates the effectiveness of CL097 in promoting proinflammatory or anti-inflammatory cytokine release and enhancing a newborn’s ability to secrete cytokines. To verify whether the CD4+ T cell number in infected mothers is correlated with the secretion of cytokines triggered by TLRs agonists, we analyzed the groups according to their total CD4+ T cell counts and divided the groups into those with <350 cells/µL and those with >350 cells/µL. The WHO guidelines recommend a threshold of <350 CD4+ T cells/µL as the criteria for beginning HAART therapy because at this level, there is substantial risk for the clinical progression of HIV. Therefore, we utilized the cutoff value of >350 CD4+ T cells/µL for the HIV-positive group. Nevertheless, all HIV-infected mothers received HAART treatment, and 40% had a nadir CD4+ T cell count of <350 cell/µL during pregnancy. Interestingly, we verified that the HIV-infected mothers only responded to CL097 stimulation when their CD4+ T cell counts were >350 cells/µL, indicating that an increase in the total number of CD4+ T cells is associated with IL-10 and TNF-α responses. This emphasizes the positive correlation between the secretion of IL-10 and TNF-α induced by CL097 activation.

This result is in agreement with a previous study showing that IL-10 secretion induced by TLR3 and TLR4 stimulation in HIV patients is associated with maintenance of the CD4+ T cell population [40]. Moreover, the secretion of IL-10 induced by CL097 activation in HIV-infected mothers could also be associated with the generation of regulatory T cells. However, further assessment is required to determine whether the induction of regulatory T cells is caused by CL097-induced IL-10 secretion.

To assess type I interferon, an important antiviral factor, we investigated the IFN-α response. Although PBMCs from HIV-infected mothers secreted less IFN-α upon TLR3, TLR7 and TLR9 activation, CL097 increased the response in CB from infected mothers compared with CB from uninfected mothers. In fact, CL097 was able to induce PBMC expression of IRF-7, IFN-α and TNF-α in CB from infected mothers to a degree similar to that of CB in the control group, in contrast to the TLR9 agonist, which had no adjuvant effect. Thus, we verified the contribution of the TLR7/8 agonist as an immunotherapeutic adjuvant. Although the transcriptional levels of IRF-7 and IFN-α in infected mothers were similar to the control group, protein secretion levels were clearly reduced, suggesting a post-translational deficit in type I IFN secretion in chronic HIV-1-infected individuals. In contrast, this effect was not observed for TNF-α mRNA and protein as demonstrated intracellularly in mDCs.

Dendritic cells are crucial in mediating innate immunity and promoting adaptive immune responses. Our findings showed that engagement of TLRs resulted in TNF-α secretion by mDCs only when they were activated by CL097 in infected mothers and CB. The effect was most likely due to TLR8 activation, as imiquimod, a ligand of TLR7, did not induce mDC activation. This finding confirms the data obtained with PBMCs showing that TLR7/8 stimulation induced robust TNF-α secretion in HIV-infected mother-newborn pairs. However, it raises the issue of whether this activation might increase productive HIV-1 replication in DCs, considering that ligation of both TLR8 and DC-SIGN is required for the induction of signal transduction pathways that promote the synthesis of complete viral transcripts from integrated proviral DNA [41] **.** Whether the use of CL097 might increase productive HIV replication in DCs remains to be determined.

In contrast to the ability of mDCs in the HIV-infected pairs to respond to CL097, pDCs were significantly impaired in both infected mothers and newborns. This result highlights the fact that chronic HIV infection induces an important dysfunction in the innate immune response. The mDCs seem to be the more flexible population when compared with pDCs in mothers infected with HIV. Untreated chronic HIV-1-infected patients under HAART recovered mDC numbers in contrast to a sustained loss of pDCs in parallel with decreased IFN-α secretion [42], [43]. The decline of pDCs inversely correlates with viral load and is associated with a decrease in the level of IFN-α production per cell and lower CD4^+^ T cell counts [44], [45]. Evidence of immune exhaustion in pDCs and a better reconstitution of pDCs in patients treated with HAART early argue that an earlier start to therapy results in preservation of pDC numbers and function [46].

Type I IFN responses are critical in the early phase of the immune response, but the chronic and systemic activation of pDCs can paradoxically lead to deleterious consequences for the immune system, including the inhibition of T cell proliferation and the promotion of cell death [47]. Although innate immune responses are highly potent in suppressing HIV-1 replication, the overactivation of pDCs inhibits antiviral T cell responses [48].

The production of IFN-α by pDCs depends on the transcription factor, IRF-7, which is expressed at high constitutive levels in pDCs, but not in other PBMC populations [49]. The transcription factor, MyD88, is required for signaling through TLR7 and TLR9, and it forms a complex with IRF-7 that is required for type I IFN production in pDCs [50]. In CB, there is defective expression of type I IFN genes in response to TLR9 and TLR7 ligation or to human cytomegalovirus or Herpes simplex 1 exposure, and this impairment in type I IFN gene expression is associated with a failure of IRF-7 to translocate [51]. Nevertheless, our findings in uninfected newborns showed an increased frequency of pDCs secreting IFN-α upon CL097, Imiquimod and CpG stimulation, achieving the same frequency as adult counterpart. Only when we assessed IFN-α secretion levels in PBMCs stimulated with CL097 did we detect a marked decrease in secretion in CB compared with the uninfected adult counterpart. It is possible that despite the similar percentage of pDCs, the IFN-α secretion per cell is reduced in newborns compared with adults. Many cells have the potential to produce IFN-α, whereas pDCs produce 10- to 1000-fold more IFN-α than other cell types [52].

Taken together, our results show a dysfunctional innate immune response in HIV-1-infected mothers and newborns. The activation of TLR7/TLR8 could restore defective cytokine secretion by mDCs, but not by pDCs. Whether defective pDC activation during pregnancy prevents overactivation of the immune system or is due to signaling defects has yet to be clarified. Applying this knowledge of the innate immune response of HIV-infected mothers and newborns could contribute to new formulations for drugs or the development of novel vaccination strategies.

## Materials and Methods

### Ethics Statement

The research involving human participants reported in this study was approved by the São Paulo University Institutional Use Committee. The research was conducted according to the Declaration of Helsinki. We obtain informed consent from mothers on the behalf of the newborn participants involved in the study. All study subjects provided written informed consent under the approval of the São Paulo University Institutional Ethics Committee.

### Study Population

The study enrolled 15 HIV-infected mothers from the obstetrics outpatient clinic at HC-FMUSP and 15 HIV-1 uninfected mothers from the São Paulo University Maternity Hospital. The median age was 29.5 years (range 17–38) for HIV-infected mothers and 29 years (18–33) for uninfected mothers. All HIV-1-infected mothers were given AZT prophylactically and received intravenous AZT 3–5 h before cesarean section. At the time of delivery, 40 mL of peripheral blood from the umbilical cord and a sample of peripheral blood from the mother were collected. All mothers had negative serology for syphilis, hepatitis B and C, toxoplasmosis and other diseases. Uninfected mothers were also negative for HIV and gave birth by caesarean section.

An infant was categorized as uninfected if the infant had two negative HIV-1 RNA PCR assay results at separate visits. Infants not meeting these criteria were classified as having an indeterminate infection status and were excluded from the study.

### Reagents

Ligands of TLR2 (Pam3Cks4), TLR3 (poly I:C), TLR4 (lipopolysaccharide from *Streptococcus minnesota*), TLR7 (Imiquimode-R837) and TLR7/TLR8 (CL097) were purchased from Invivogen (San Diego, CA, USA). The ligand for TLR9 (oligodeoxynucleotides-CpG) was synthesized by Eurofins MWG Operon (Huntsville, AL, USA).

### Cell Culture

PBMCs were isolated from heparinized venous blood from the mother and from cord blood by Ficoll-Hypaque gradient centrifugation (GE Healthcare Bio-Sciences AB, Uppsala, Sweden) and diluted in RPMI medium supplemented with 5% AB human serum (RPMI-S) (Sigma, St. Louis, MO, USA). Cultures of PBMCs (2.0×10^5^ cells/well) were incubated in 96-well plates (Costar, Cambridge, MA, USA) in medium with or without ligands for TLR2 (Pam3Cks4, 0.5 µg/mL), TLR3 (poly I:C, 1 µg/mL), TLR4 (LPS, 1.25 µg/mL), TLR7 (Imiquimode, 1.25 µg/mL), TLR7/TLR8 (CL097, 2.5 µg/mL) and TLR9 (CpG, 2 µM/mL) for 48 h at 37°C and 5% CO_2_. Cell-free supernatants were harvested and stored at −80°C until cytokine measurements were taken by cytometric bead array.

### Cytokine Measurements

Supernatants obtained from PBMC cultures were measured for TNF-α, IFN-α and IL-10 using a cytometric bead array (Becton Dickinson, San Diego, CA, USA) and analyzed by flow cytometry (FACS Calibur, BD, San Jose, CA, USA).

### Flow Cytometry

We assessed TNF*-*α and IFN-α secretion in mDCs and pDCs, respectively, induced by TLR7, TLR7/8 and TLR9 stimulation in PBMCs from mothers and cord blood samples. PBMCs (2 x 10^6^ cells/500 µL) were cultured in 48-well plates (Costar) in RPMI-S with imiquimode (1.25 µg/mL), CL097 (5 µg/mL), or CpG (2 µM/mL) for 20 h at 37°C and 5% CO_2_. Brefeldin A (10 µg/mL, Sigma) was added to cells 5 h after the beginning of the culture. Cells were then washed in phosphate buffered saline (PBS) containing 0.5% bovine serum albumin (BSA) and 0.1% sodium azide, and pDCs were characterized as Lin^neg^/CD123+ using a FITC–lineage 1 cocktail (Lin1, a mix of CD3, CD14, CD16, CD19, CD20 and CD56), APC-HLA-DR and PECy5-CD123 (BD Pharmingen, San Jose, CA). For mDC characterization, we used PECy5-CD11c, FITC-lineage 1 and APC-HLA-DR. All antibodies were from BD Biosciences (San Jose, CA, USA). Cells were fixed and permeabilized with buffer (Fix & Perm A and B, Invitrogen, Grand Island, NY, USA) and stained with antibodies to PE-IFN-α (BD Pharmingen) for pDCs or PE-TNF-α for mDCs. Cells not stained were used as control to set the gates. After incubation, the cells were washed once in PBS/0.5% BSA, processed by flow cytometry (FACS Calibur, BD) and analyzed with Flowjo software (Tree Star, Inc., Ashland, OR, USA).

### Real-time PCR

PBMCs were cultured with the TLR7/TLR8 agonist (CL097, 2.5 µg/mL) or TLR9 agonist (CpG, 2 µM/mL) for 4 h at 37°C and 5% CO_2_. Total RNA from PBMCs was extracted using the RNeasy Plus Mini kit (Qiagen, Valencia, CA, USA), and reverse transcription was performed with a Sensiscript Reverse Transcriptase kit (Qiagen). Real-time PCR was performed with the following primers: IRF-7 (sense 5′-TGGTCCTGGTGAAGCTGGAA-3′, antisense 5′-GATGTCGTCATAGAGG CTGTTG-3′), IFN-α (sense 5′-GACTCCATCTTGGCTGTGA-3′, antisense 5′-TGA TTTCTGCTCTGACAACCT-3′) and TNF-α (sense 5′-CCCAGGCAGTCAGA TCAT CTTC-3′, antisense 5′-GCTTGAGGGTTTGCTACAACATG-3′). Glyceraldehyde-3-phosphate dehydrogenase (GAPDH) mRNA levels in all samples in the same well were analyzed to normalize the mRNA content using the following primers: sense 5′-GAAGGTGAAGGTCGGAGT-3′ and antisense 5′-GAAGATGGTGATGGGATTTC-3′. To ensure the exclusion of genomic DNA, each sample was tested in a reaction without reverse transcriptase. PCR was performed in an Applied 7300 machine using specific cytokine primers and SYBR green (Applied Biosystems) fluorescence detection for 10 minutes at 95°C followed by 40 cycles of 15 seconds each at 95°C and 60 seconds at 60°C. The specificity of the reaction was examined by a dissociation curve. Analysis of the amplification results were visualized and analyzed using the SDS System software (Applied Biosystems). Relative quantitative expression was calculated as described [53].

### Statistical Analysis

The Mann-Whitney *U* test was used to compare variables between HIV-infected mothers and healthy controls or between cord blood samples from infected and uninfected mothers. The Wilcoxon test, a non-parametric paired test, was used to compare the difference between gene expression levels under basal and stimulated conditions. *P*≤0.05 was considered significant.

## Supporting Information

Figure S1 **Correlation between TLR7/8 activation and TNF-α and IL-10 secretion (A, B) and type of TLR activation and TNF-α secretion (C, D) in HIV-1-infected (closed circles) and uninfected mothers (open circles).**
(TIF)

Figure S2 **Representative gating strategy of mDCs and pDCs in a healthy adult individual.**
(TIF)

Figure S3 **Percentage of mDCs (A) and pDCs (B) and mDC:pDC ratio (C) in HIV-1-infected mothers (M) and cord blood (CB, closed circles) and uninfected mothers and cord blood (open circles).**
(TIF)

## References

[1] (2012) UNAIDS Global Report AIDS epidemic -WHO. 1–103 p.

[2] Agangi A, Thorne C, Newell ML (2005) Increasing likelihood of further live births in HIV-infected women in recent years. BJOG 112: 881–888. 15957987 10.1111/j.1471-0528.2005.00569.x

[3] Mussi-Pinhata MM, Rego MA, Freimanis L, Kakehasi FM, Machado DM, et al (2007) Maternal antiretrovirals and hepatic enzyme, hematologic abnormalities among human immunodeficiency virus type 1-uninfected infants: the NISDI perinatal study. Pediatr Infect Dis J 26: 1032–1037. 17984811 10.1097/INF.0b013e31812f56ed

[4] Kuhn L, Kasonde P, Sinkala M, Kankasa C, Semrau K, et al (2005) Does severity of HIV disease in HIV-infected mothers affect mortality and morbidity among their uninfected infants? Clin Infect Dis 41: 1654–1661. 16267740 10.1086/498029 PMC1351118

[5] Koyanagi A, Humphrey JH, Ntozini R, Nathoo K, Moulton LH, et al (2011) Morbidity among human immunodeficiency virus-exposed but uninfected, human immunodeficiency virus-infected, and human immunodeficiency virus-unexposed infants in Zimbabwe before availability of highly active antiretroviral therapy. Pediatr Infect Dis J 30: 45–51. 21173675 10.1097/INF.0b013e3181ecbf7e

[6] Dauby N, Goetghebuer T, Kollmann TR, Levy J, Marchant A (2012) Uninfected but not unaffected: chronic maternal infections during pregnancy, fetal immunity, and susceptibility to postnatal infections. Lancet Infect Dis 12: 330–340. 22364680 10.1016/S1473-3099(11)70341-3

[7] Lohman-Payne B, Slyker J, Rowland-Jones SL (2010) Immune-based approaches to the prevention of mother-to-child transmission of HIV-1: active and passive immunization. Clin Perinatol 37: 787–805, ix. 10.1016/j.clp.2010.08.005 PMC2998888 21078451

[8] Dolcini G, Derrien M, Chaouat G, Barre-Sinoussi F, Menu E (2003) Cell-free HIV type 1 infection is restricted in the human trophoblast choriocarcinoma BeWo cell line, even with expression of CD4, CXCR4 and CCR5. AIDS Res Hum Retroviruses 19: 857–864. 14585217 10.1089/088922203322493021

[9] Rowland-Jones SL, Nixon DF, Aldhous MC, Gotch F, Ariyoshi K, et al (1993) HIV-specific cytotoxic T-cell activity in an HIV-exposed but uninfected infant. Lancet 341: 860–861. 8096564 10.1016/0140-6736(93)93063-7

[10] Kuhn L, Meddows-Taylor S, Gray G, Tiemessen C (2002) Human immunodeficiency virus (HIV)-specific cellular immune responses in newborns exposed to HIV in utero. Clin Infect Dis 34: 267–276. 11740717 10.1086/338153

[11] Legrand FA, Nixon DF, Loo CP, Ono E, Chapman JM, et al (2006) Strong HIV-1-specific T cell responses in HIV-1-exposed uninfected infants and neonates revealed after regulatory T cell removal. PLoS One 1: e102. 17183635 10.1371/journal.pone.0000102 PMC1762312

[12] Mold JE, Michaelsson J, Burt TD, Muench MO, Beckerman KP, et al (2008) Maternal alloantigens promote the development of tolerogenic fetal regulatory T cells in utero. Science 322: 1562–1565. 19056990 10.1126/science.1164511 PMC2648820

[13] Warning JC, McCracken SA, Morris JM (2011) A balancing act: mechanisms by which the fetus avoids rejection by the maternal immune system. Reproduction 141: 715–724. 21389077 10.1530/REP-10-0360

[14] Adkins B, Leclerc C, Marshall-Clarke S (2004) Neonatal adaptive immunity comes of age. Nat Rev Immunol 4: 553–564. 15229474 10.1038/nri1394

[15] Diebold SS, Kaisho T, Hemmi H, Akira S, Reis e Sousa C (2004) Innate antiviral responses by means of TLR7-mediated recognition of single-stranded RNA. Science 303: 1529–1531. 14976261 10.1126/science.1093616

[16] Lund JM, Alexopoulou L, Sato A, Karow M, Adams NC, et al (2004) Recognition of single-stranded RNA viruses by Toll-like receptor 7. Proc Natl Acad Sci U S A 101: 5598–5603. 15034168 10.1073/pnas.0400937101 PMC397437

[17] Meier A, Altfeld M (2007) Toll-like receptor signaling in HIV-1 infection: a potential target for therapy? Expert Rev Anti Infect Ther 5: 323–326. 17547495 10.1586/14787210.5.3.323

[18] Reis e Sousa C (2004) Toll-like receptors and dendritic cells: for whom the bug tolls. Semin Immunol 16: 27–34. 14751761 10.1016/j.smim.2003.10.004

[19] Pasare C, Medzhitov R (2004) Toll-like receptors and acquired immunity. Semin Immunol 16: 23–26. 14751760 10.1016/j.smim.2003.10.006

[20] de Brito CA, Goldoni AL, Sato MN (2009) Immune adjuvants in early life: targeting the innate immune system to overcome impaired adaptive response. Immunotherapy 1: 883–895. 20636030 10.2217/imt.09.38

[21] Futata EA, Fusaro AE, de Brito CA, Sato MN (2012) The neonatal immune system: immunomodulation of infections in early life. Expert Rev Anti Infect Ther 10: 289–298. 22397563 10.1586/eri.12.9

[22] Levy O (2007) Innate immunity of the newborn: basic mechanisms and clinical correlates. Nat Rev Immunol 7: 379–390. 17457344 10.1038/nri2075

[23] Angelone DF, Wessels MR, Coughlin M, Suter EE, Valentini P, et al (2006) Innate immunity of the human newborn is polarized toward a high ratio of IL-6/TNF-alpha production in vitro and in vivo. Pediatr Res 60: 205–209. 16864705 10.1203/01.pdr.0000228319.10481.ea

[24] Caron JE, La Pine TR, Augustine NH, Martins TB, Hill HR (2010) Multiplex analysis of toll-like receptor-stimulated neonatal cytokine response. Neonatology 97: 266–273. 19955831 10.1159/000255165

[25] Levy O, Suter EE, Miller RL, Wessels MR (2006) Unique efficacy of Toll-like receptor 8 agonists in activating human neonatal antigen-presenting cells. Blood 108: 1284–1290. 16638933 10.1182/blood-2005-12-4821 PMC1895876

[26] Smed-Sorensen A, Lore K, Vasudevan J, Louder MK, Andersson J, et al (2005) Differential susceptibility to human immunodeficiency virus type 1 infection of myeloid and plasmacytoid dendritic cells. J Virol 79: 8861–8869. 15994779 10.1128/JVI.79.14.8861-8869.2005 PMC1168781

[27] Desai S, Chaparro A, Liu H, Haslett P, Arheart K, et al (2007) Impaired CCR7 expression on plasmacytoid dendritic cells of HIV-infected children and adolescents with immunologic and virologic failure. J Acquir Immune Defic Syndr 45: 501–507. 17468665 10.1097/QAI.0b013e3180654811

[28] Velilla PA, Montoya CJ, Hoyos A, Moreno ME, Chougnet C, et al (2008) Effect of intrauterine HIV-1 exposure on the frequency and function of uninfected newborns' dendritic cells. Clin Immunol 126: 243–250. 18201932 10.1016/j.clim.2007.11.004

[29] Morgado JM, Pratas R, Laranjeira P, Henriques A, Crespo I, et al (2008) The phenotypical and functional characteristics of cord blood monocytes and CD14(−/low)/CD16(+) dendritic cells can be relevant to the development of cellular immune responses after transplantation. Transpl Immunol 19: 55–63. 18346638 10.1016/j.trim.2007.11.002

[30] Willems F, Vollstedt S, Suter M (2009) Phenotype and function of neonatal DC. Eur J Immunol 39: 26–35. 19137537 10.1002/eji.200838391

[31] Charrier E, Cordeiro P, Cordeau M, Dardari R, Michaud A, et al (2012) Post-transcriptional down-regulation of Toll-like receptor signaling pathway in umbilical cord blood plasmacytoid dendritic cells. Cell Immunol 276: 114–121. 22578600 10.1016/j.cellimm.2012.04.010

[32] Mogensen TH, Melchjorsen J, Larsen CS, Paludan SR (2010) Innate immune recognition and activation during HIV infection. Retrovirology 7: 54. 20569472 10.1186/1742-4690-7-54 PMC2904714

[33] Burlingham WJ (2009) A lesson in tolerance–maternal instruction to fetal cells. N Engl J Med 360: 1355–1357. 19321873 10.1056/NEJMcibr0810752 PMC2886144

[34] Lester RT, Yao XD, Ball TB, McKinnon LR, Kaul R, et al (2008) Toll-like receptor expression and responsiveness are increased in viraemic HIV-1 infection. AIDS 22: 685–694. 18356597 10.1097/QAD.0b013e3282f4de35

[35] Benito JM, Lopez M, Lozano S, Ballesteros C, Martinez P, et al (2005) Differential upregulation of CD38 on different T-cell subsets may influence the ability to reconstitute CD4+ T cells under successful highly active antiretroviral therapy. J Acquir Immune Defic Syndr 38: 373–381. 15764953 10.1097/01.qai.0000153105.42455.c2

[36] Rosenblatt HM, Stanley KE, Song LY, Johnson GM, Wiznia AA, et al (2005) Immunological response to highly active antiretroviral therapy in children with clinically stable HIV-1 infection. J Infect Dis 192: 445–455. 15995958 10.1086/431597

[37] Brenchley JM, Price DA, Schacker TW, Asher TE, Silvestri G, et al (2006) Microbial translocation is a cause of systemic immune activation in chronic HIV infection. Nat Med 12: 1365–1371. 17115046 10.1038/nm1511

[38] Marchetti G, Bellistri GM, Borghi E, Tincati C, Ferramosca S, et al (2008) Microbial translocation is associated with sustained failure in CD4+ T-cell reconstitution in HIV-infected patients on long-term highly active antiretroviral therapy. AIDS 22: 2035–2038. 18784466 10.1097/QAD.0b013e3283112d29

[39] Levy O, Zarember KA, Roy RM, Cywes C, Godowski PJ, et al (2004) Selective impairment of TLR-mediated innate immunity in human newborns: neonatal blood plasma reduces monocyte TNF-alpha induction by bacterial lipopeptides, lipopolysaccharide, and imiquimod, but preserves the response to R-848. J Immunol 173: 4627–4634. 15383597 10.4049/jimmunol.173.7.4627

[40] Villacres MC, Kono N, Mack WJ, Nowicki MJ, Anastos K, et al (2012) Interleukin 10 responses are associated with sustained CD4 T-cell counts in treated HIV infection. J Infect Dis 206: 780–789. 22693231 10.1093/infdis/jis380 PMC3491747

[41] Gringhuis SI, van der Vlist M, van den Berg LM, den Dunnen J, Litjens M, et al (2010) HIV-1 exploits innate signaling by TLR8 and DC-SIGN for productive infection of dendritic cells. Nat Immunol 11: 419–426. 20364151 10.1038/ni.1858

[42] Chehimi J, Campbell DE, Azzoni L, Bacheller D, Papasavvas E, et al (2002) Persistent decreases in blood plasmacytoid dendritic cell number and function despite effective highly active antiretroviral therapy and increased blood myeloid dendritic cells in HIV-infected individuals. J Immunol 168: 4796–4801. 11971031 10.4049/jimmunol.168.9.4796

[43] Pacanowski J, Kahi S, Baillet M, Lebon P, Deveau C, et al (2001) Reduced blood CD123+ (lymphoid) and CD11c+ (myeloid) dendritic cell numbers in primary HIV-1 infection. Blood 98: 3016–3021. 11698285 10.1182/blood.v98.10.3016

[44] Barron MA, Blyveis N, Palmer BE, MaWhinney S, Wilson CC (2003) Influence of plasma viremia on defects in number and immunophenotype of blood dendritic cell subsets in human immunodeficiency virus 1-infected individuals. J Infect Dis 187: 26–37. 12508143 10.1086/345957

[45] Donaghy H, Gazzard B, Gotch F, Patterson S (2003) Dysfunction and infection of freshly isolated blood myeloid and plasmacytoid dendritic cells in patients infected with HIV-1. Blood 101: 4505–4511. 12576311 10.1182/blood-2002-10-3189

[46] Kamga I, Kahi S, Develioglu L, Lichtner M, Maranon C, et al (2005) Type I interferon production is profoundly and transiently impaired in primary HIV-1 infection. J Infect Dis 192: 303–310. 15962225 10.1086/430931

[47] Heikenwalder M, Polymenidou M, Junt T, Sigurdson C, Wagner H, et al (2004) Lymphoid follicle destruction and immunosuppression after repeated CpG oligodeoxynucleotide administration. Nat Med 10: 187–192. 14745443 10.1038/nm987

[48] Boasso A, Royle CM, Doumazos S, Aquino VN, Biasin M, et al (2011) Overactivation of plasmacytoid dendritic cells inhibits antiviral T-cell responses: a model for HIV immunopathogenesis. Blood 118: 5152–5162. 21931112 10.1182/blood-2011-03-344218 PMC3217402

[49] Izaguirre A, Barnes BJ, Amrute S, Yeow WS, Megjugorac N, et al (2003) Comparative analysis of IRF and IFN-alpha expression in human plasmacytoid and monocyte-derived dendritic cells. J Leukoc Biol 74: 1125–1138. 12960254 10.1189/jlb.0603255

[50] Honda K, Ohba Y, Yanai H, Negishi H, Mizutani T, et al (2005) Spatiotemporal regulation of MyD88-IRF-7 signalling for robust type-I interferon induction. Nature 434: 1035–1040. 15815647 10.1038/nature03547

[51] Danis B, George TC, Goriely S, Dutta B, Renneson J, et al (2008) Interferon regulatory factor 7-mediated responses are defective in cord blood plasmacytoid dendritic cells. Eur J Immunol 38: 507–517. 18200500 10.1002/eji.200737760

[52] Siegal FP, Kadowaki N, Shodell M, Fitzgerald-Bocarsly PA, Shah K, et al (1999) The nature of the principal type 1 interferon-producing cells in human blood. Science 284: 1835–1837. 10364556 10.1126/science.284.5421.1835

[53] Livak KJ, Schmittgen TD (2001) Analysis of relative gene expression data using real-time quantitative PCR and the 2(T)(-Delta Delta C) method. Methods 25: 402–408. 11846609 10.1006/meth.2001.1262

