# Supplementary figures and images for: TLR7/TLR8 Activation Restores Defective Cytokine Secretion by Myeloid Dendritic Cells but Not by Plasmacytoid Dendritic Cells in HIV-Infected Pregnant Women and Newborns
Source: PLoS One. 2013 Jun 27;8(6):e67036. doi: 10.1371/journal.pone.0067036 (PMC3694931; doi:10.1371/journal.pone.0067036)

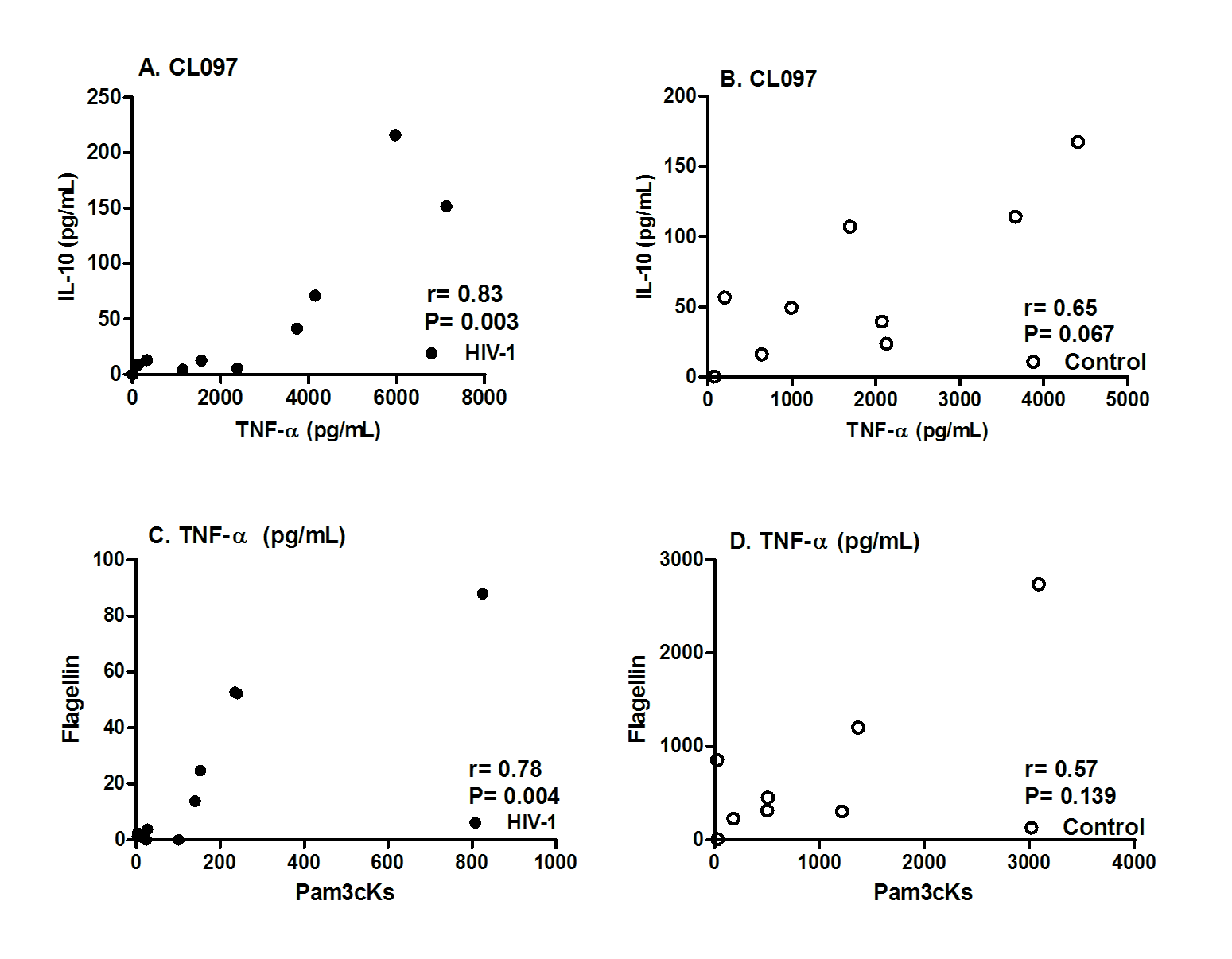

Supplement: Figure S1 — Correlation between TLR7/8 activation and TNF-α and IL-10 secretion (A, B) and type of TLR activation and TNF-α secretion (C, D) in HIV-1-infected (closed circles) and uninfected mothers (open circles). (TIF) [file pone.0067036.s001.tif]

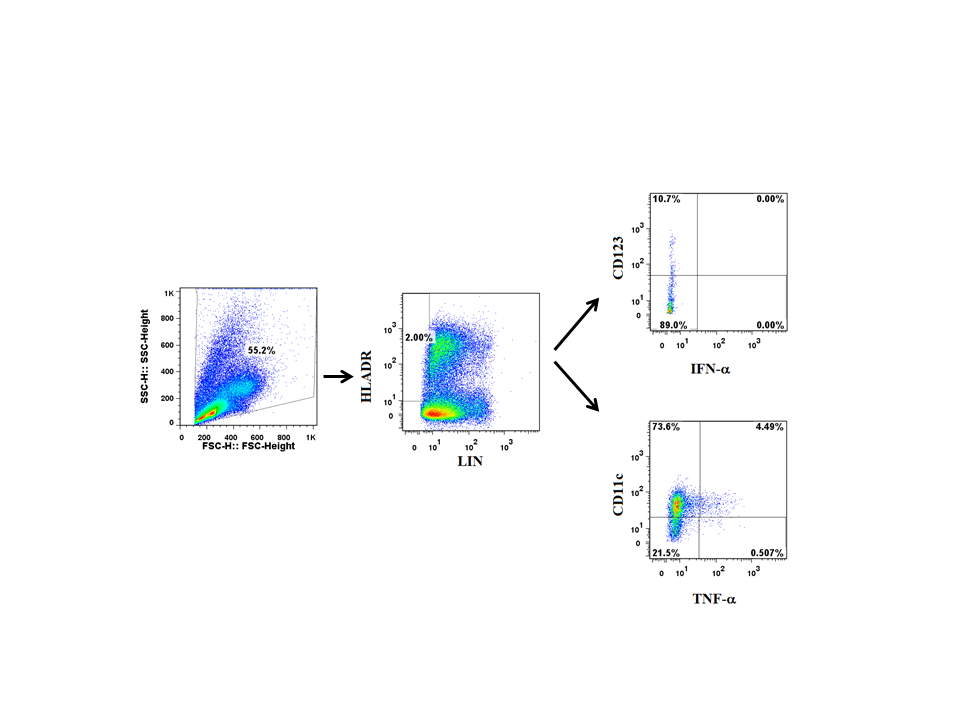

Supplement: Figure S2 — Representative gating strategy of mDCs and pDCs in a healthy adult individual. (TIF) [file pone.0067036.s002.tif]

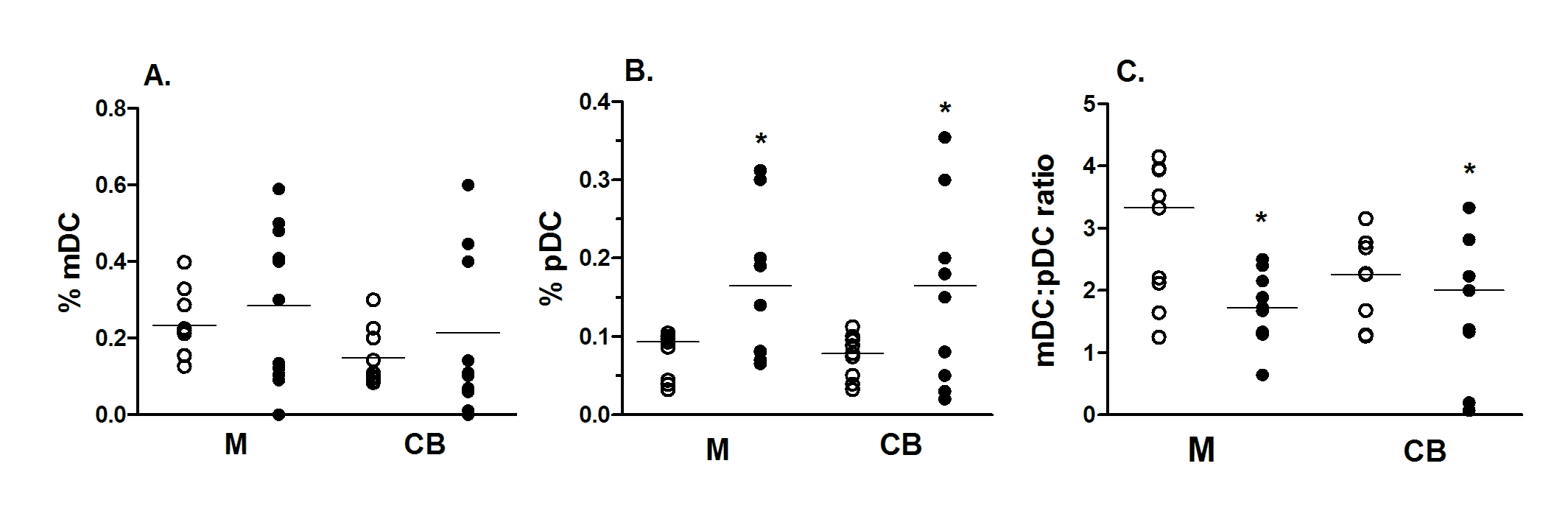

Supplement: Figure S3 — Percentage of mDCs (A) and pDCs (B) and mDC:pDC ratio (C) in HIV-1-infected mothers (M) and cord blood (CB, closed circles) and uninfected mothers and cord blood (open circles). (TIF) [file pone.0067036.s003.tif]
